# Fucosylated N-glycans as early biomarkers of COVID-19 severity

**DOI:** 10.3389/fimmu.2023.1204661

**Published:** 2023-06-05

**Authors:** Beatrix Paton, Pol Herrero, Joaquim Peraire, Antoni del Pino, Silvia Chafino, Javier Martinez-Picado, Fréderic Gómez-Bertomeu, Anna Rull, Núria Canela, Manuel Suárez

**Affiliations:** ^1^ Eurecat, Centre Tecnològic de Catalunya, Centre for Omic Sciences (Joint Unit Eurecat- Universitat Rovira i Virgili), Unique Scientific and Technical Infrastructure (ICTS), Reus, Spain; ^2^ Hospital Universitari de Tarragona Joan XXIII (HJ23), Tarragona, Spain; ^3^ Institut Investigació Sanitària Pere Virgili (IISPV), Tarragona, Spain; ^4^ Centro de Investigación Biomédica en Red de Enfermedades Infecciosas (CIBERINFEC), Instituto de Salud Carlos III, Madrid, Spain; ^5^ Universitat Rovira i Virgili (URV), Tarragona, Spain; ^6^ IrsiCaixa AIDS Research Institute, Badalona, Spain; ^7^ Germans Trias i Pujol Research Institute (IGTP), Badalona, Spain; ^8^ University of Vic-Central University of Catalonia (UVic-UCC), Vic, Spain; ^9^ Catalan Institution for Research and Advanced Studies (ICREA), Barcelona, Spain; ^10^ Universitat Rovira i Virgili, Departament de Bioquímica i Biotecnologia, Nutrigenomics Research Group, Tarragona, Spain

**Keywords:** N-glycosylation, fucosylation, biomarker, COVID-19, LC-MS/MS

## Abstract

**Background:**

The pathological mechanisms of SARS-CoV-2 in humans remain unclear and the unpredictability of COVID-19 progression may be attributed to the absence of biomarkers that contribute to the prognosis of this disease. Therefore, the discovery of biomarkers is needed for reliable risk stratification and to identify patients who are more likely to progress to a critical stage.

**Methods:**

Aiming to identify new biomarkers we analysed N-glycan traits in plasma from 196 patients with COVID-19. Samples were classified into three groups according to their severity (mild, severe and critical) and obtained at diagnosis (baseline) and at 4 weeks of follow-up (postdiagnosis), to evaluate their behaviour through disease progression. N-glycans were released with PNGase F and labelled with Rapifluor-MS, followed by their analysis by LC-MS/MS. The Simglycan structural identification tool and Glycostore database were employed to predict the structure of glycans.

**Results:**

We determined that plasma from SARS-CoV-2-infected patients display different N-glycosylation profiles depending on the disease severity. Specifically, levels of fucosylation and galactosylation decreased with increasing severity and Fuc1Hex5HexNAc5 was identified as the most suitable biomarker to stratify patients at diagnosis and distinguish mild from critical outcomes.

**Conclusion:**

In this study we explored the global plasma glycosignature, reflecting the inflammatory state of the organs during the infectious disease. Our findings show the promising potential of glycans as biomarkers of COVID-19 severity.

## 1 Introduction

Three significant coronavirus outbreaks have been documented to date, with the most recent being caused by the 2019 novel coronavirus (2019-nCoV, also known as SARS-CoV-2), which is known to cause the Coronavirus Disease-2019 (COVID-19) (1). Since the start of the pandemic in 2020, over 6.6 million deaths from COVID-19 have been reported globally, resulting in one of the major global health crises of the 21^st^ century (2). In most cases, SARS-CoV-2 infection is accompanied by a variety of symptoms, including fever, cough, and general malaise (3). Acute lung injury and acute respiratory distress syndrome appear in more severe COVID-19 instances, which can induce morbidity and mortality due to pneumonia and inflammation caused by damage to the alveolar lumen (4, 5).

The pathophysiology of COVID-19 still needs to be better understood, which will lead to better clinical and therapeutic approaches for patients, improved management of healthcare resources, as well as advancements in vaccination strategy. The SARS-CoV-2 vaccinations that are currently available have been crucial for controlling the pandemic, but their long-term effectiveness and delivery methods are still being tested (6, 7). Additionally, it remains unclear if an association exists between having certain risk factors and an increased death rate, and why some COVID-19 patients can fight the infection while others require hospitalization. Therefore, prognostic and predictive biomarkers must be identified to promptly detect patients who are more likely to evolve to a critical state of the disease (8, 9).

Within this framework, omics datasets, such as proteomics, lipidomics and metabolomics have been used to improve the understanding of the immunopathogenesis and the host immune response to SARS-CoV-2, and study the biological factors that contribute to a worse prognosis of patients with COVID-19 (9–11). In addition to these omics techniques, the detection of glycomic alterations could potentially extend the knowledge of COVID-19. Plasma glycomic changes are considered biomarkers for a variety of illnesses, including diabetes, systemic lupus erythematosus, colorectal cancer, and cardiovascular disease (12–16). Beyond being used as a biomarker, the circulating glycome on plasma has been shown to mediate and drive significant immunological functions (17). Plasma glycoproteins are released from organs and enter the circulation by active secretion or leakage. Several studies have shown that glycosylation of such circulating glycoproteins can reflect the inflammatory states of these organs during chronic diseases (18). The study of the glycoprofile in COVID-19 has received some attention, specifically in serum immunoglobulin Gs (IgGs). Afucosylated Fc N-glycans in IgGs specific for SARS-CoV-2-spike protein have been found to be more prevalent in critically ill patients (19), and total IgG N-glycome composition has been reported to be different between mild and severe disease patients (20). Additionally, levels of galactose and sialic acid structures on IgGs have been reported to predict the development of a poor COVID-19 disease (21).

Samples were collected during the first and second waves of the COVID-19 pandemic in Spain, which started in March 2020 and October 2020, respectively. During this period, alpha and beta (lineages B.1.1.7 and B.1.351, respectively) were the predominant circulating variants (22) and patients were still not vaccinated against COVID-19, as vaccines were not made available until later in the year (23). This cohort was initially used in a previous study in which metabolomics, lipidomics and proteomics analyses were conducted to identify key molecules involved in the mechanistic pathways of the disease. Specific molecules related to complement and coagulation cascades, platelet activation, cell adhesion, acute inflammation, energy production (Krebs cycle and Warburg effect), amino acid catabolism and lipid transport were identified as fingerprints of the acute disease. Additionally, fetuin-A, inter-α-trypsin, glutamic acid and cholesteryl ester 18:0 were collectively proposed as a novel panel of biomarkers to differentiate mild from critical COVID-19 outcomes (9). To enrich the knowledge about COVID-19 progression and extend the list of potential biomarkers, our study focused on analysing the total plasma N-glycome composition of 196 patients at diagnosis and at 4 weeks postdiagnosis, to assess if it can help stratify COVID-19 patients and evaluate whether it can act as a biomarker for the development of COVID-19. We aimed to obtain a global plasma glycosignature, instead of exclusively focusing on the IgG N-glycome, to reflect the inflammatory state of the organs during the infectious disease.

## 2 Results

The sample size employed in this study consisted of 196 COVID-19 patients, classified into three groups according to their severity (mild, severe and critical) (9). Disease progression was evaluated at 4 weeks postdiagnosis for 122 of the aforementioned patients. Firstly, the glycosignature in COVID-19 patients was presented, followed by a comparison between groups of severity at diagnosis and an evaluation of potential glycosignatures to indicate disease severity in COVID-19 at diagnosis. Additionally, the N-glycan profile at 4 weeks postdiagnosis was evaluated to explore the potential of total plasma N-glycome to act as a biomarker for COVID-19 disease progression. N-glycan compositions detected by mass spectrometry were reported as follows: Hex [hexose, either galactose (Gal), or mannose (Man)], HexNAc [N-acetylhexosamine], Fuc [fucose] and Neu5Ac [N-acetylneuraminic acid].

### 2.1 N-linked glycosylation variations among COVID-19 patients at diagnosis

**Figure 1:**
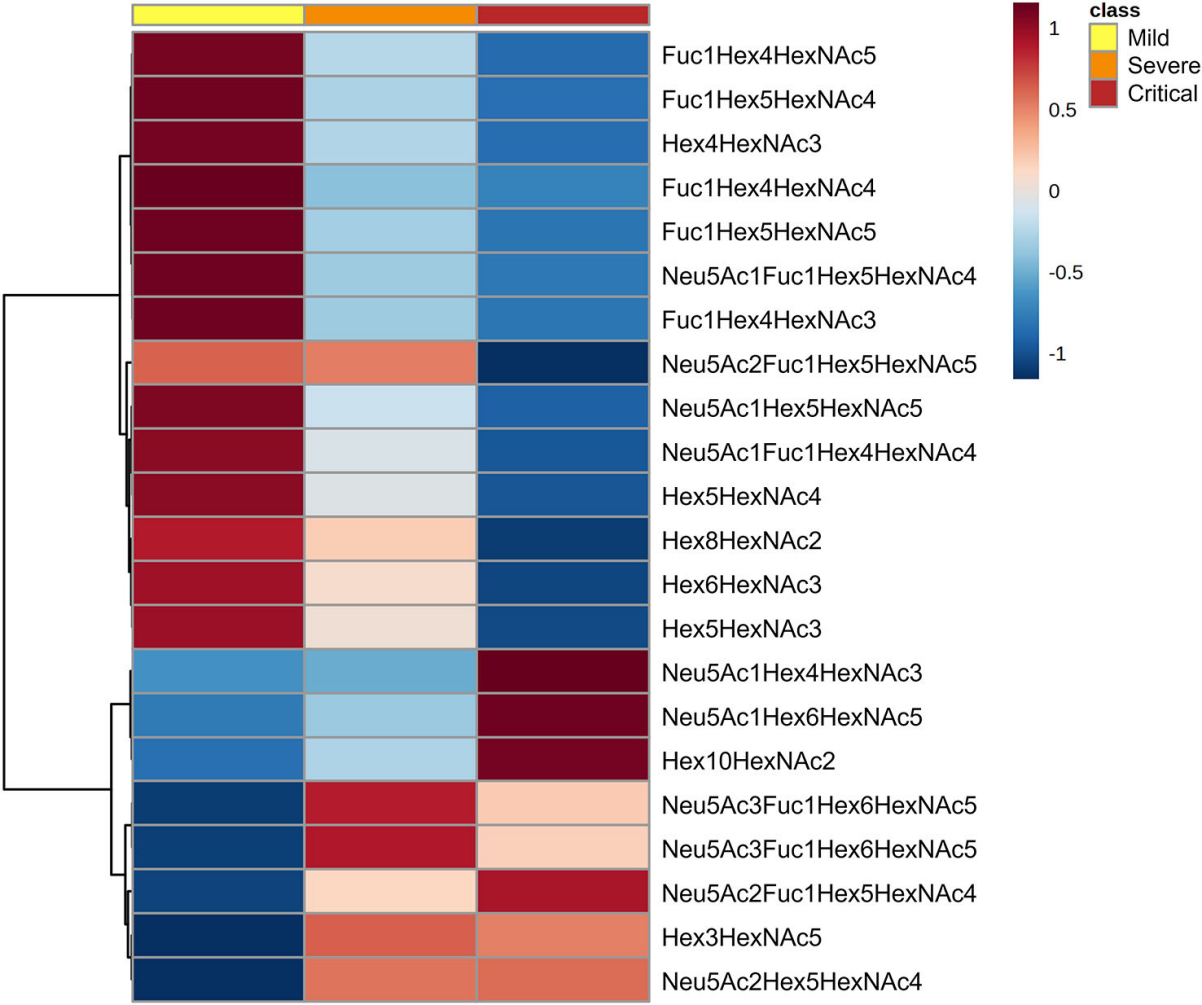
N-glycan signatures associated with COVID-19 disease severity. Heatmap plotting the significant relative abundance of N-glycans increasing or decreasing at diagnosis in accordance with disease severity. Significant differences (p < 0.05) between mild, severe and critical COVID‐19 groups of patients were determined by Kruskal-Wallis test. Columns indicate the degree of disease severity: mild (left), severe (centre) and critical (right) groups. Mean values for each compound in each COVID‐19 group (columns) are colour‐coded based on relative abundance, low (blue) and high (red).

The N-glycan profile at diagnosis was evaluated to explore the prognostic potential of total plasma N-glycome as a biomarker for COVID-19 disease severity. Patients were classified at diagnosis as mild (n=56), severe (n=105) and critical (n=35) (24). Total plasma N-glycan composition was determined by LC-MS/MS analysis of RFMS-labelled glycans, confirming structures with MS/MS data. A total of 36 structures were identified in all patients (**Supplementary Table 1**) and 13 additional structures were detected but could not be accurately annotated. The most abundant structures in all the samples were biantennary and were specifically Neu5Ac2Hex5HexNAc4 followed by Neu5Ac1Hex5HexNAc4 and Fuc1Hex3HexNAc4. A total of 22 N-glycans were found to be statistically significant between groups at diagnosis (**Figure 1**). These 22 N-glycans were further tested as potential biomarkers for COVID-19 for their ability to predict the disease course at diagnosis.

### 2.2 Plasma N-glycosylation profile at diagnosis aids in the stratification of patients and predicts COVID-19 prognosis

**Figure 2:**
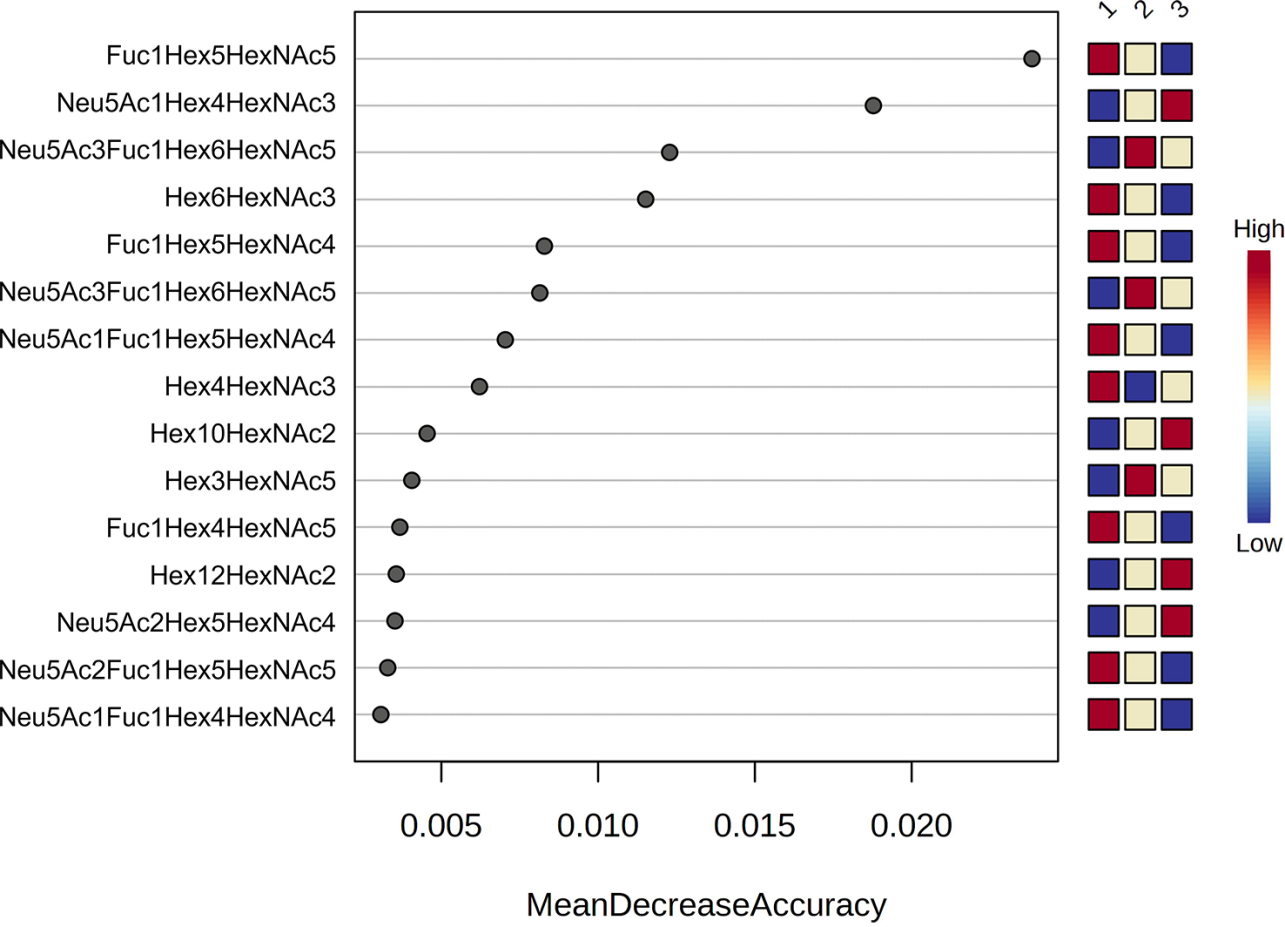
Random forest analysis. Random forest modelling of the top 15 significant N-glycans with the highest discriminatory power between groups (1 = mild, 2 = severe and 3 = critical). Coloured boxes on the right indicate the relative concentrations of the corresponding N-glycan in each group.

A random forest analysis was used to evaluate the effectiveness of the statistically significant total plasma N-glycans as predictive biomarkers to indicate disease severity in COVID-19 at diagnosis. Results indicated that the top 3 N-glycans with the highest discriminatory power between groups were Fuc1Hex5HexNAc5 and Hex6HexNAc3, which decrease with increasing severity, and Neu5Ac1Hex4HexNAc3, which increases with severity (**Figure 2**).

**Figure 3:**
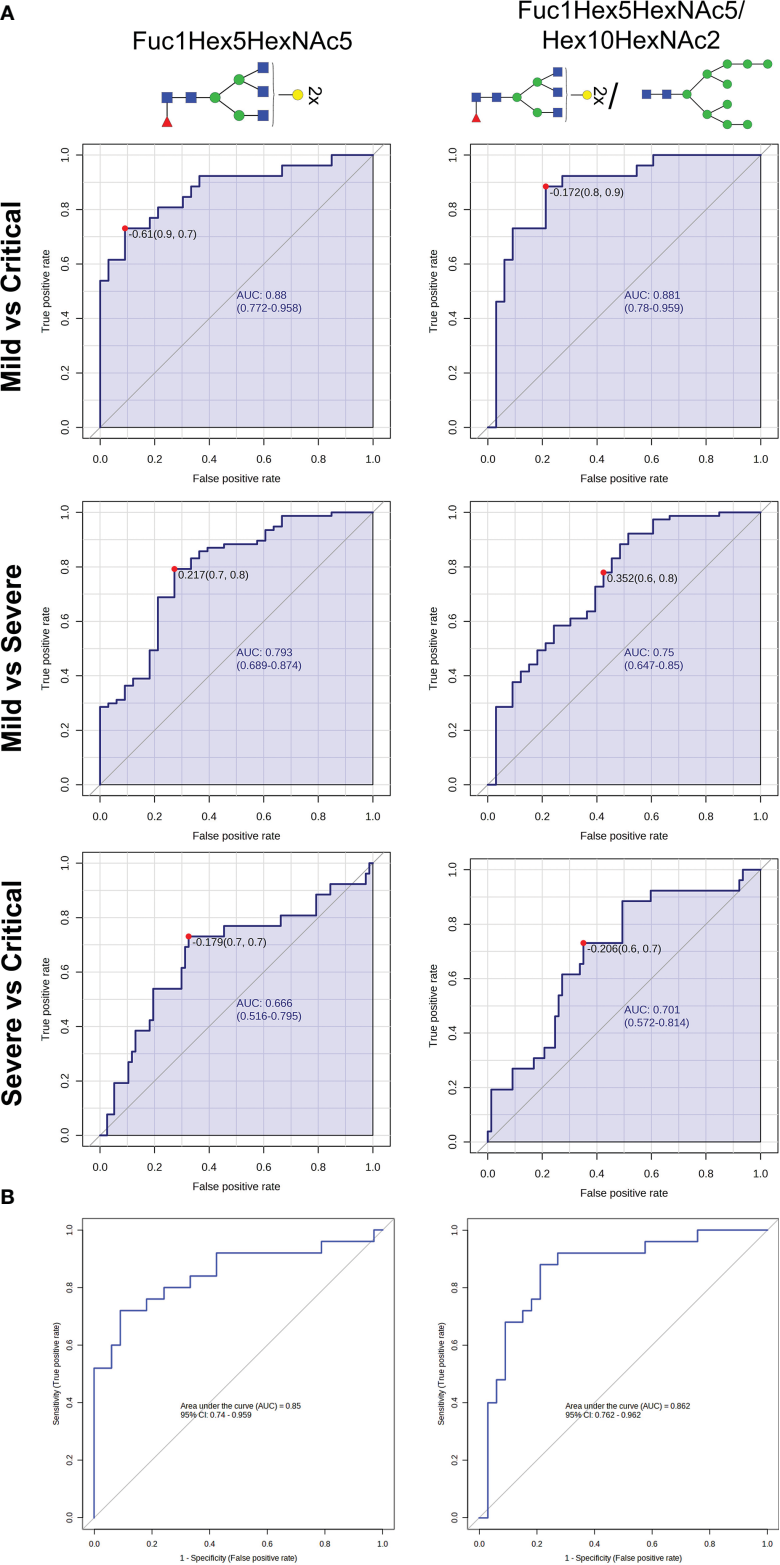
N-glycan biomarkers to indicate COVID-19 severity at diagnosis. **(A)** Receiver operating characteristic (ROC) curves analyses of Fuc1Hex5HexNAc5 and Fuc1Hex5HexNAc5/Hex10HexNAc2 for distinguishing COVID-19 patients between groups of severity. **(B)** Binary logistic regression modelling analysis testing the accuracy of Fuc1Hex5HexNAc5 and Fuc1Hex5HexNAc5/Hex10HexNAc2 to differentiate mild from critically ill patients with COVID-19 in a randomly selected set of patients. For the N-glycan cartoons, green circles denote mannose, yellow circles denote galactose, blue squares denote N-acetylglucosamine, red triangles denote fucose, and purple diamonds denote N-acetylneuraminic acid.

Subsequently, prediction models were built to evaluate the predictive efficacy of some of the N-glycans with the highest classification accuracy. Receiver operating characteristic (ROC) curves were generated through a binary logistic regression analysis comparing the mild *vs*. critical groups, severe *vs*. critical groups and mild *vs*. severe groups (**Table 1**; **Figure 3**). The ROC curve analysis was performed for an individual N-glycan and for the ratio of two N-glycans. The ratio was computed as it was able to provide more information than the two corresponding N-glycans alone. These ratios were computed for all possible N-glycan pairs and the top-ranked ratio (based on p-values) was selected for further biomarker analysis.

**Table 1:** ROC curve analysis data obtained for a selected N-glycan and a ratio between two selected N-glycans showing the area under the curve (AUC) scores, significance, specificity and sensitivity.

COVID-19 group	N-glycan/s	AUC	Significance	Sensitivity (%)	Specificity (%)
**Mild *vs* Critical**	Fuc1Hex5HexNAc5	0.880	<0.001	73.1	90.9
	Fuc1Hex5HexNAc4/Hex10HexNAc2	0.881	<0.001	88.5	78.8
**Severe *vs* Critical**	Fuc1Hex5HexNAc5	0.793	<0.001	77.9	57.6
	Fuc1Hex5HexNAc4/Hex10HexNAc2	0.750	<0.001	79.2	72.7
**Mild *vs* Severe**	Fuc1Hex5HexNAc5	0.666	0.02	73.1	67.5
	Fuc1Hex5HexNAc4/Hex10HexNAc2	0.701	0.002	73.1	64.9

The ratio of Fuc1Hex5HexNAc4/Hex10HexNAc2 was the top-ranked ratio (p<0.001), showing a high capacity to differentiate between mild and critical patients, with an AUC of 0.881 and a specificity and a sensitivity of 90.9 and 73.1%, respectively. Similarly, Fuc1Hex5HexNAc5, individually, was also optimal to distinguish between mild from critical patients (AUC = 0.88; p<0.001) (**Table 1**; **Figure 3A**). In the random forest analysis, Fuc1Hex5HexNAc5 was considered to be the N-glycan with the highest classification accuracy.

Regarding the other two pairwise comparisons, severe *vs* critical and mild *vs* severe, more biomarkers should be considered to rapidly identify the clinical progression of patients at an early stage, as AUCs ranged between 0.666 and 0.793. Next, a regression model was performed in a randomly selected set of patients to validate the accuracy of these aforementioned N-glycans to properly predict mild and critical outcomes at diagnosis. Results indicated that the ratio Fuc1Hex5HexNAc4/Hex10HexNAc2 (AUC=0.862, p<0.001, specificity=78.8% and sensitivity=88.0%) and Fuc1Hex5HexNAc5 (AUC=0.85, p<0.001, specificity=90.9% and sensitivity=72.0%) could be suitable biomarkers to distinguish mild from critical outcomes (**Figure 3B**).

### 2.3 N-linked glycosylation variations among COVID-19 patients at 4 weeks postdiagnosis

**Figure 4:**
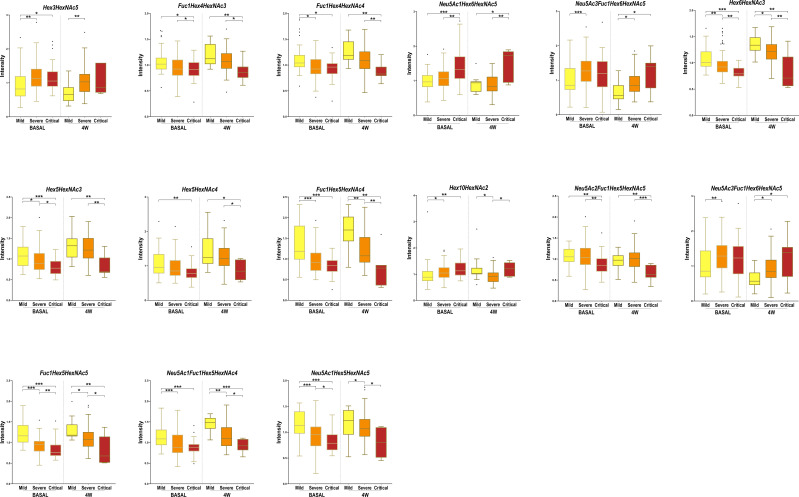
Box-and-whisker plots showing N-glycan abundance levels in COVID-19 patients. Relative abundance of total plasma N-glycans in different COVID-19 severities (mild, severe and critical) at diagnosis (BASAL) and at 4 weeks postdiagnosis (4W). Kruskal-Wallis, *p-value < 0.05; **p-value < 0.01 ***p-value < 0.001.

The N-glycan profile at 4 weeks postdiagnosis was evaluated to explore the potential of total plasma N-glycome to act as a biomarker for COVID-19 disease course prediction. A total of 18 N-glycans were found to be statistically significant between groups at 4 weeks postdiagnosis, 15 of which were also significantly altered at diagnosis. The progression of these 15 N-glycans at diagnosis and at 4 weeks for the different groups of severity is shown in **Figure 4**. The aforementioned N-glycans (Fuc1Hex5HexNAc5 and Hex10HexNAc2) that were tested as potential biomarkers using a regression model were altered at diagnosis and at 4 weeks postdiagnosis. Fuc1Hex5HexNAc5 showed the same tendency at both time points, decreasing with increasing severity. Differently, Hex10HexNAc2 increased with increasing severity at diagnosis and at 4 weeks postdiagnosis. Interestingly, for both N-glycans, the significance of the alterations was higher at diagnosis than at 4 weeks. The remaining 13 N-glycans altered at both time points showed similar tendencies at diagnosis and at 4 weeks postdiagnosis (**Figure 4**). More specifically, all the statistically significant fucosylated structures decreased with increasing severity at diagnosis and at 4 weeks, except for Neu5Ac3Fuc1Hex6HexNAc5 which increased with increasing severity.

**Figure 5:**
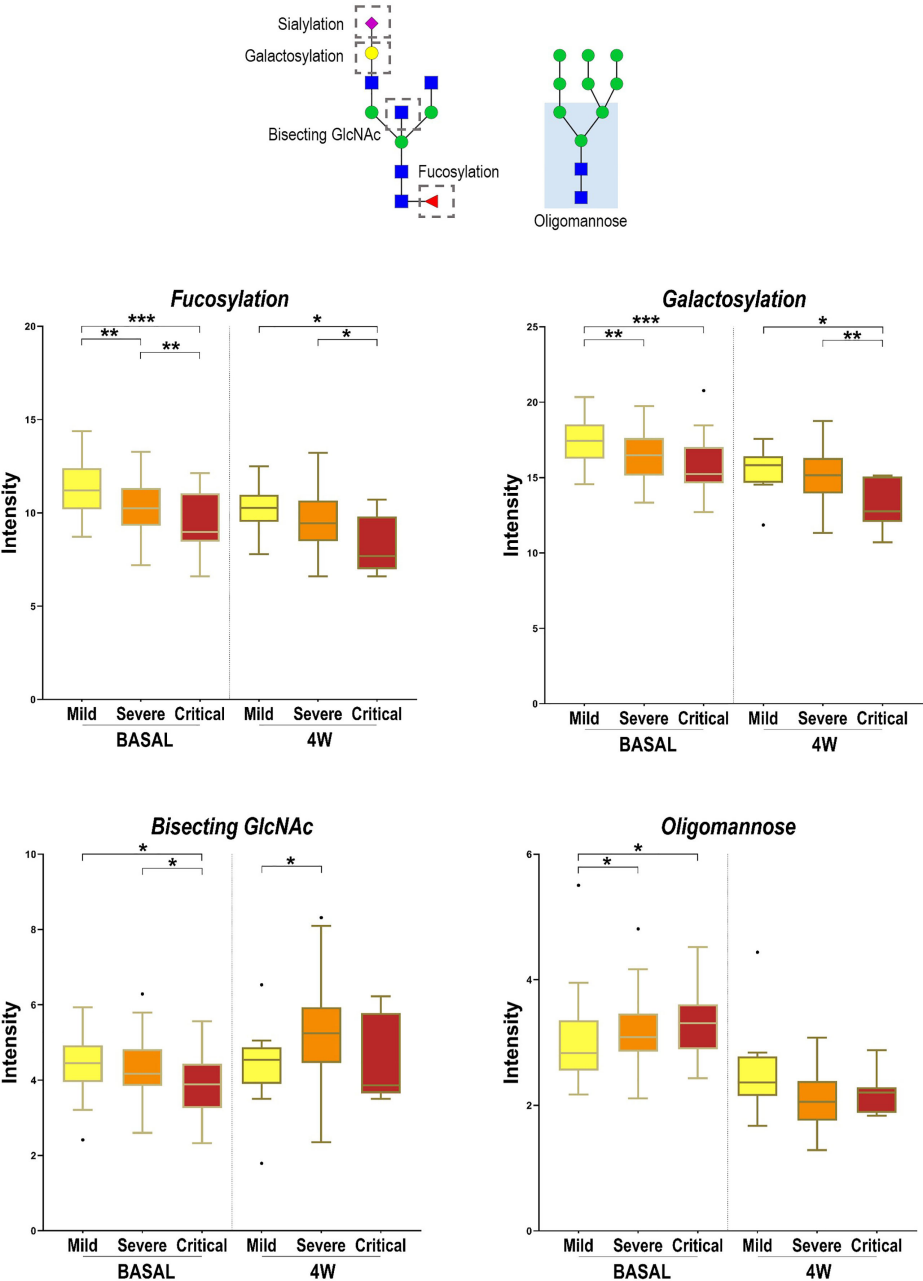
Alterations in four N-glycan families according to disease severity. Relative abundance of total Bisecting GlcNAc, fucosylation, galactosylation and oligomannose structures in different COVID-19 severities (mild, severe and critical) at diagnosis (BASAL) and at 4 weeks postdiagnosis (4W). Kruskal-Wallis, *p-value < 0.05; **p-value < 0.01; ***p-value < 0.001. For the N-glycan cartoons, green circles denote mannose, yellow circles denote galactose, blue squares denote N-acetylglucosamine, red triangles denote fucose, and purple diamonds denote N-acetylneuraminic acid.

Three additional N-glycans were altered only at 4 weeks between severity groups, specifically Hex4HexNAc5 and NeuAc1Hex4HexNAc5, which increased with severity and Hex7HexNAc2, which decreased with severity. To obtain a global picture of the observed significant alterations at diagnosis and at 4 weeks postdiagnosis, glycan structures were grouped by their traits in terms of bisecting GlcNAc (N-acetylglucosamine), oligomannose-type, fucosylation, galactosylation and sialylation (**Figure 5**). Total plasma bisecting GlcNAc structures significantly decreased in critical patients when compared with mild and severe ones at diagnosis, whereas the trend shifted at 4 weeks postdiagnosis, increasing in severe patients compared to mild ones. A similar pattern was observed for fucosylation and galactosylation, both decreasing with increasing severity at diagnosis and at 4 weeks postdiagnosis. Notably, a greater significance between groups of severity was observed at diagnosis than at 4 weeks. Differently, oligomannose structures increased with severity at diagnosis and showed no significant alterations between groups at 4 weeks postdiagnosis. No significant changes were observed in sialylation.

## 3 Discussion

We have demonstrated that total plasma from SARS-CoV-2-infected patients displays different glycosylation profiles that can be detected at diagnosis and at 4 weeks postdiagnosis, aiding in the stratification of patients and with potential to predict COVID-19 prognosis. A total of 15 N-glycans were statistically significant between groups at diagnosis as well as at 4 weeks postdiagnosis, showing a similar trend at both time points. We determined that fucosylated and galactosylated structures significantly decreased in critical patients when compared with mild and severe ones. In line with our results, a deficiency of IgG galactosylation has been observed in COVID-19 patients (25) that display a poor disease course (21), showing that agalactosylation on IgG is associated with the activation of the lectin-initiated complement pathway in the development of inflammatory diseases (25, 26). Moreover, we found that agalactosylated structures were increased in critical patients compared to moderate and mild ones. Levels of galactosylation in IgG are, in fact, one of the most prominent glycosylation alterations observed in several chronic inflammatory and autoimmune diseases (27). Regarding fucosylation, recent studies on IgG glycome show a decrease in fucosylation in SARS-CoV-2 infected patients, which upregulates antibody-dependent cell cytotoxicity (ADCC) in acute immune responses (25). Also, in accordance with our results at diagnosis, glycans with bisecting GlcNAc have been reported to be significantly lower in COVID-19 cases, compared to controls, potentially causing a decrease in the modulation of the inflammatory response among COVID-19 patients (25). Loss of sialylation in total IgG Fc has been observed in severe patients compared to mild ones (21, 25). The ability of IgG to participate in complement-dependent cytotoxicity (CDC) activity *via* C1q binding is decreased in the absence of sialylation, increasing the activation of the lectin-initiated alternative complement pathway (28). In our study, some sialylated N-glycans increased with severity and others decreased with severity at diagnosis. No significant differences in sialylation were reported at 4 weeks postdiagnosis. With the obtention of a global plasma glycosignature, we potentially observed the inflammatory state of the organs during the disease. The tendencies observed were similar to those reported in the IgG N-glycome, indicating that the IgG N-glycan profile can be obtained without purifying IgG, thus simplifying the sample preparation.

Other prognostic biomarkers have been proposed for COVID-19 based on metabolomics, lipidomics and proteomics analyses (9, 29–31). In a previous study from this same cohort of patients, fetuin-A, inter-α-trypsin inhibitor 3, glutamic acid and cholesterol ester 18:0 were reported to be the most accurate biomarkers of the critical clinical progression of COVID-19 (9). The addition of Fuc1Hex5HexNAc5 or Fuc1Hex5HexNAc4/Hex10HexNAc2 to this set of biomarkers could potentially improve the prediction of mild and critical outcomes. Moreover, these glycans were significantly altered between groups at diagnosis and at 4 weeks postdiagnosis, showing the same tendency at both time points. Interestingly, for these N-glycans, the significance of the alterations was higher at diagnosis than at 4 weeks, showing the potential of these glycans to become biomarkers that can be detected at early stages.

It is crucial to account for potential confounding factors that can influence the interpretation of the results. We specifically looked for the influence of pre-existing health conditions that could impact COVID-19 severity and affect biomarker levels. A few patients in the cohort presented comorbidities such as diabetes, cardiovascular disease or cancer. We observed that N-glycans Hex3HexNAc5 and Hex4HexNAc5 were affected by these comorbidities. However, most of the significantly altered N-glycans between groups of severity, including all fucosylated N-glycans, were not biased by the presence of other comorbidities. Importantly, no associations were observed between any underlying medical conditions and the proposed biomarkers, Fuc1Hex5HexNAc5 and Fuc1Hex5HexNAc4/Hex10HexNAc2. Future studies should be designed to minimise the impact of confounding factors. The validation of the obtained results using larger and well-characterised multicentric cohorts will help account for population heterogeneity and increase the generalisability of findings. These studies will also help to determine the impact of different SARS-CoV-2 variants in total plasma glycosylation, as in the moment of sample recruitment the circulating variants were mainly alpha and beta. Additionally, future longitudinal studies including analysis of samples before and after infection would be useful to determine whether these structures could be employed as preinfection biomarkers to predict the critical clinical progression of COVID-19. However, it is important to note the difficulty implied in obtaining a sample from an individual before and after the SARS-CoV-2 infection. On the other hand, the analysis of samples from healthy volunteers, who have never been diagnosed with the disease, could aid in the identification of biomarkers that are highly specific to COVID-19 infection, as well as help control for confounding factors that may be present in COVID-19 positive patients. Finally, additional studies in the vaccinated population should focus on breakthrough infections requiring hospitalization, as well as measuring the effectiveness of vaccines to prevent infection and hospitalization. Overall, this study reveals a novel risk screening system based on the plasma N-glycome signature, able to assess the stratification of COVID-19 patients and discriminate the progression of the disease at diagnosis. The reported minimally invasive blood biomarker has the potential to improve and optimise the management of healthcare resources and vaccination strategies in COVID-19.

## 4 Methods

### 4.1 Study design and classification criteria

A total of 196 patients with SARS-CoV-2 infection who had positive polymerase chain reaction (PCR) confirmation within the first 21 days of infection make up the COVID-19 patient cohort. Patients were categorised into 3 groups of severity (mild (n=56), severe (n=105) and critical (n=35)), according to the inclusion criteria described in “Diagnosis and Treatment Protocol for COVID-19 Patients (version 8 trial)” (24). Additionally, disease progression was evaluated at 4 weeks postdiagnosis for 122 of the aforementioned patients (mild (n=22), severe (n=82) and critical (n=18)). The severity classification changed for 49 patients during the 4 weeks postdiagnosis, with 16 patients changing from mild to severe, 32 from severe to critical and one from critical to severe. Data from patients were stored in a database with information about the hospitalization, including the symptoms that were present at the time of admission, radiological findings, the severity of pneumonia, the need for oxygen therapy, the medical treatment received, as well as demographic information and previously diagnosed diseases of interest (9). Patients’ demographic and relevant clinical data are summarised in **Supplementary Table 2**. Briefly, the cohort median age increased with COVID-19 severity and the female sex predominated in the mild and critical groups. Hypertension was the most common comorbidity in all groups and more predominant in severe and critical patients. Drug administration and the need for oxygen and aggressive treatments were consistent with the severity of the disease. A total of 25 patients from the cohort died from COVID-19. The serum biochemical composition was also characterised at the time of admission in the entire cohort, including both routine and inflammatory parameters. The most significant differences in the blood pattern were observed between mild and critical patients (9).

### 4.2 Samples recruitment

The sampling protocol consisted of a clinical evaluation, blood cell count, and standard biochemical parameters at inclusion (baseline). Serum samples were stored at -80°C at BioBank - Institut d’Investigació Sanitària Pere Virgili (IISPV) facilities until analysed (9).

### 4.3 Ethics

Protocols were carried out in accordance with the recommendations of the Ethical and Scientific Committees from each participating institution and were approved by the Committee for Ethical Clinical Research following the rules of Good Clinical Practice from the IISPV (079/2020, CEIm IISPV). The CEIm IISPV is an independent committee, which oversees the correct adherence to the ethical standards governing clinical trials and research projects that are carried out in our environment, specifically in terms of its methodology, ethics and laws. Its members include both health and non-health professionals. All subjects or their relatives gave written informed consent in accordance with the Declaration of Helsinki (9).

### 4.4 Reagents

Ammonium bicarbonate (ABC), ammonium formate and formic acid (LC-MS grade) were purchased from Sigma-Aldrich (St. Louis, MO, USA). Acetonitrile (LC-MS grade) was purchased from Merck (Darmstadt, Germany). The water used throughout the study was purified with a Milli-Q system from Millipore (Burlington, MA, USA).

### 4.5 De-N-glycosylation and labelling of N-glycans

The release and labelling of N-glycans were done using a method previously described (32). Briefly, total protein was quantified with the Bradford assay. Sample denaturation, de-N-glycosylation, labelling with Rapifluor-MS (RFMS), and purification were performed in accordance with the Waters Corporation “GlycoWorks RapiFluor-MS N-Glycan Kit Care and Use Manual” (p/n 715004793). Briefly, 15 µg of protein were denatured at 90°C for 3 min in the presence of 5% (w/v) RapiGest. De-*N*-glycosylation was then conducted at 50°C for 5 min, by adding 1.2 μL of Rapid PNGase F. Subsequently, the digested samples were directly labelled with 6 μL of RFMS reagent without the need for purification. The reaction proceeded at room temperature for 5 min before using a GlycoWorks μElution Plate for the SPE Clean-up procedure. Glycans were eluted with 200 mM ammonium acetate in 5% acetonitrile.

### 4.6 LC-MS/MS

The LC-MS/MS analysis was carried out using a previously described analytical method (32). Derivatised samples were analysed using an Agilent UHPLC 1290 Infinity Series coupled to an Agilent qTOF/MS 6550 Series (Agilent Technologies, Santa Clara, CA). N-glycans were separated on a Waters ACQUITY UPLC BEH amide column (2.1 mm × 150 mm i.d., particle size 1.7 μm), using 50 mM ammonium formate solution (mobile phase A) and 100% acetonitrile (mobile phase B). The analysis was performed at a flow rate of 0.4 mL/min, with an injection volume of 20 µL, and the column temperature set at 60°C. The chromatographic gradient began by ramping mobile phase A from 25% to 46% over a period of 35 minutes. From 35 to 36.5 min, the gradient ramped from 46 to 100% solvent A and the flow rate was lowered to 0.2 mL/min. 100% solvent A was held constant from 36.5 to 39.5 min, after which the percentage of solvent A decreased to 25%, from 39.5 min to 43.1 min. The flow rate was then increased back to 0.4 mL/min from 43.1 to 47.6 min and solvent A was held constant at 25%. Lastly, the parameters were held constant from 47.6 to 55.0 min. The qTOF operated in positive electrospray ionisation mode (ESI+), and mass spectra were recorded between m/z 300–1700 at 1.5 spectra/s. The source conditions were set as follows: nebuliser gas at 25 psi, gas temperature at 200°C, gas flow at 12 L/min, sheath gas temperature at 250°C, sheath gas flow at 12 L/min, capillary voltage at 3500 V, and nozzle voltage at 500 V. Additionally, tandem mass experiments (MS/MS) using data dependence acquisition at a collision energy of 30 eV from the 10 most intense ions were used for identification purposes.

### 4.7 Data processing of LC-MS data

The MS data were first processed using Agilent MassHunter Qualitative Analysis B.07 software. For the identification of N-glycans, total ion chromatograms (TIC) containing MS/MS fragmentation data were deconvoluted using the “find molecular feature” algorithm, which detected chromatographic peaks considered to be N-glycans. The resulting list of entities containing the MS/MS data was exported and loaded to Simglycan software for molecular and structural elucidation. Simglycan is a high-throughput structural identification tool that uses a built-in database with theoretical fragmentation profiles to predict the structure of glycans (33). Additionally, the Simglycan results were matched against the GlycoStore database (https://www.glycostore.org) to refine the identification results. GlycoStore provided elution property information for over 850 unique structures including standardised retention times, expressed as glucose units (GU), for the RFMS-labelled glycans (34). Following the identification process, 36 N-glycans were identified in human plasma and 13 additional structures were detected but could not be annotated. Subsequently, the exact masses [M+H]^+^, [M+2H]^2+^ or [M+3H]^3+^ of these structures were extracted on all samples using Agilent Mass Hunter Quantitative software (B.07) to generate a refined matrix of quantitative data for statistical purposes. Finally, the obtained data matrix, which included the peak area for each identified N-glycan, was normalised by dividing the peak area of each N-glycan by the sum of the peak areas of all N-glycans within each respective sample.

### 4.8 Statistical analysis

Non-parametric Kruskal-Wallis test was used to assess significant differences between groups of severity, employing Mass Profiler Professional (MPP) software v.15.1 (Agilent Technologies, Massachusetts, USA). Graphical representations were generated with GraphPad Prism software (version 9.0, GraphPad Inc., San Diego, CA) and Metaboanalyst 5.0. Both SPSS (version 21.0, SPSS Inc., Chicago, IL) and Metaboanalyst were employed to perform random forest analyses, and create binary logistic regression models and ROC curves to evaluate the potential accuracy of the selected biomarkers for predicting COVID-19 severity. Kruskal-Wallis test and χ2 test were used to determine the demographic and clinical features differences between groups of severity. The Spearman’s correlation coefficient, used to measure the association between the study cohort variables and groups of severity, was performed with SPSS. Results were considered statistically significant at p < 0.05.

## Data availability statement

The datasets presented in this study can be found in online repositories. The names of the repository/repositories and accession number(s) can be found here: GPST000341 (Glycopost).

## Ethics statement

The studies involving human participants were reviewed and approved by the Ethics Research Committee (CEIM Institut d’Investigació Sanitària Pere Virgili). The patients/participants provided their written informed consent to participate in this study.

## Author contributions

Conception and design of the study, PH, NC, AR and MS. Methodology, BP, JP, AdP, SC, JM-P and FG-B. Formal Analysis, BP and AdP. Writing – Original Draft Preparation, BP. Writing – Review and Editing, BP, PH, NC, AR and MS. Supervision, PH, NC, AR and MS. Funding Acquisition, AR and PH. All authors contributed to the article and approved the submitted version.
