# Supplementary material for: Fucosylated N-glycans as early biomarkers of COVID-19 severity
Source: Front Immunol. 2023 Jun 5;14:1204661. doi: 10.3389/fimmu.2023.1204661 (PMC10278543; doi:10.3389/fimmu.2023.1204661)
Supplement: Supplementary file 1 [file Table_1.docx]

Supplementary Material

Fucosylated N-glycans as early biomarkers of COVID-19 severity

**Beatrix Paton^1^, Pol Herrero^1^, Joaquim Peraire^2,3,4,5^, Antoni del Pino^1^, Silvia Chafino^2,3,4^, Javier Martinez-Picado^4,6,7,8,9^, Fréderic Gómez-Bertomeu^2,3,4,5^, Anna Rull* ^2,3,4,5^, Núria Canela *^1^, Manuel Suárez^10^**

*** Correspondence:** Núria Canela [nuria.canela@eurecat.org](mailto:nuria.canela@eurecat.org) ; Anna Rull [anna.rull@iispv.cat](mailto:anna.rull@iispv.cat)

**Supplementary Table 1**. N-glycan profile in COVID-19 patients. Hex, hexose (either galactose (Gal), or mannose (Man)); Fuc, fucose; HexNAc, N-acetylhexosamine; Neu5Ac, N-acetylneuraminic acid.

| **Glycan composition** | **m/z** | **Glycan Mass** |
| --- | --- | --- |
| Fuc1Hex3HexNAc4 | 887.867 | 1462.539 |
| Fuc1Hex4HexNAc3 | 867.353 | 1421.512 |
| Fuc1Hex4HexNAc4 | 968.894 | 1624.592 |
| Fuc1Hex4HexNAc5 | 1070.433 | 1827.671 |
| Fuc1Hex5HexNAc4 | 1049.920 | 1786.645 |
| Fuc1Hex5HexNAc5 | 1151.458 | 1989.721 |
| Hex10HexNAc2 | 1178.946 | 2044.697 |
| Hex3HexNAc4 | 814.838 | 1316.481 |
| Hex3HexNAc5 | 916.377 | 1519.559 |
| Hex4HexNAc2 | 692.784 | 1072.374 |
| Hex4HexNAc3 | 794.325 | 1275.456 |
| Hex4HexNAc4 | 895.864 | 1478.533 |
| Hex5HexNAc2 | 773.812 | 1234.429 |
| Hex5HexNAc3 | 875.350 | 1437.506 |
| Hex5HexNAc4 | 976.890 | 1640.586 |
| Hex6HexNAc2 | 854.838 | 1396.482 |
| Hex6HexNAc3 | 956.382 | 1599.569 |
| Hex7HexNAc2 | 935.864 | 1558.533 |
| Hex8HexNAc2 | 1016.890 | 1720.585 |
| Hex9HexNAc2 | 1097.917 | 1882.639 |
| Neu5Ac1Fuc1Hex4HexNAc3 | 1012.901 | 1712.607 |
| Neu5Ac1Fuc1Hex4HexNAc4 | 1114.440 | 1915.685 |
| Neu5Ac1Fuc1Hex5HexNAc4 | 1195.468 | 2077.741 |
| Neu5Ac1Hex4HexNAc3 | 939.872 | 1566.549 |
| Neu5Ac1Hex4HexNAc4 | 1041.411 | 1769.627 |
| Neu5Ac1Hex5HexNAc4 | 1122.439 | 1931.683 |
| Neu5Ac1Hex5HexNAc5 | 1297.007 | 2280.819 |
| Neu5Ac1Hex6HexNAc3 | 1101.924 | 1890.653 |
| Neu5Ac1Hex6HexNAc5 | 1305.003 | 2296.811 |
| Neu5Ac2Fuc1Hex5HexNAc4 | 1341.015 | 2368.835 |
| Neu5Ac2Fuc1Hex5HexNAc5 | 1442.561 | 2571.927 |
| Neu5Ac2Hex5HexNAc4 | 1267.987 | 2222.779 |
| Neu5Ac2Hex6HexNAc5 | 1450.556 | 2587.917 |
| Neu5Ac3Fuc1Hex6HexNAc5 | 1113.091 | 3025.068 |
| Neu5Ac3Hex6HexNAc5 | 1596.105 | 2879.015 |
| NeuAc1Hex5HexNAc3 | 1020.898 | 1728.601 |

**Supplementary Table 2**. Demographic and clinical features of COVID-19 study cohort.

| Variables | COVID-19 group | | | |
| --- | --- | --- | --- | --- |
|  | **Mild (n=56)** | **Severe (n=105)** | **Critical (n=35)** | **P value** |
| Male | 28 (50.0) | 71 (67.6) | 20 (67.7) | n.s. |
| Age, years | 52.0 (39.3-63.8) | 66.0 (51.5-66.0) | 64.0 (53.0-75.0) | <0.001 |
| Comorbidities – no. (%) | | | | |
| Obesity | 10 (17.9) | 27 (25.8) | 10 (28.6) | <0.001 |
| Metabolic syndrome | 0 (0) | 7 (6.7) | N.D. | <0.001 |
| Diabetes mellitus | 9 (16.1) | 22 (21.0) | 7 (20.0) | <0.001 |
| Hypertension | 16 (28.6) | 57 (54.3) | 16 (45.7) | 0.008 |
| Cardiovascular disease | 5 (8.9) | 14 (13.3) | 6 (17.1) | <0.001 |
| COPD | 2 (3.6) | 16 (15.2) | 5 (14.3) | <0.001 |
| Cancer | 5 (8.9) | 12 (11.4) | 3 (8.6) | <0.001 |
| HIV | 0 (0) | 1 (1.0) | 1 (2.9) | <0.001 |
| COVID-19 Symptoms | | | | |
| Fever | 32 (57.1) | 74 (70.5) | 26 (74.3) | <0.001 |
| Cough | 24 (42.9) | 66 (62.9) | 22 (62.9) | 0.021 |
| Fatigue | 14 (25.0) | 30 (28.6) | 10 (28.6) | <0.001 |
| Dyspnea | 19(33.9) | 64 (61.0) | 29 (82.9) | 0.021 |
| Anosmia | 10 (17.9) | 10 (9.5) | 2 (5.7) | <0.001 |
| Oxygen therapy and intensive care | | | | |
| Oxygen required | 7(12.5) | 57 (54.3) | 30(85.7) | <0.001 |
| Low-flow oxygen administration  (Ventimask or nasal prongs) | 4(7.1) | 34 (32.4) | 5 (14.3) | <0.001 |
| High-flow oxygen administration/NIMV | 0(0) | 20 (19.0) | 4 (11.4) | <0.001 |
| MV/intubation | 2(3.6) | 15(14.3) | 21 (60.0) | <0.001 |
| Vasopressors or dialysis required | 2(3.6) | 7 (6.7) | 8 (22.9) | <0.001 |
| Mortality | | | | |
| Exitus | 2 (3.6) | 14 (13.3) | 9 (25.7) | <0.001 |
| Medicines subscribed | | | | |
| Hydroxychloroquine | 3 (5.4) | 16 (15.2) | 6 (17.1) | <0.001 |
| Azithromycin | 15 (26.8) | 57 (54.3) | 7 (20.0) | 0.007 |
| Lopinavir/ritonavir | 1 (1.8) | 13 (12.4) | 5 (14.3) | <0.001 |
| Tocilizumab | 0 (0) | 12 (11.4) | 5 (14.3) | <0.001 |
| Interferon | 0 (0) | 0 (0) | 1 (2.9) | <0.001 |
| Corticosteroids | 13 (23.2) | 74 (70.5) | 18 (51.4) | <0.001 |
| Remdesivir | 3 (5.4) | 34 (32.4) | 8 (22.9) | <0.001 |

Data are presented as n (%) or median (interquartile range: 25-75). P values comparing mild, severe and critical patients were computed using non-parametric Kruskal-Wallis test for continuous data and χ2 test for categorical data. P value < 0.05 was considered significant. COPD, chronic obstructive pulmonary disease; NIMV, non-invasive mechanical ventilation; MV, mechanical ventilation.
